# Bone Mineral Density Loss After Kidney Transplantation: Metabolic Determinants, Diagnostic Approaches, and Preventive Strategies

**DOI:** 10.1016/j.ekir.2026.106685

**Published:** 2026-06-30

**Authors:** Clemens Untersulzner, Markus Pirklbauer, Pieter Evenepoel, Heinz Zoller, Andreas Kronbichler

**Affiliations:** 1 Department of Internal Medicine IV, Nephrology and Hypertension, Medical University of Innsbruck, Innsbruck, Austria; 2 Department of Microbiology, Immunology and Transplantation, Nephrology and Renal Transplantation Research Group, Katholieke Universiteit Leuven, Leuven, Belgium; 3 Department of Nephrology and Renal Transplantation, University Hospitals Leuven, Leuven, Belgium; 4 Department of Medicine I, Gastroenterology, Hepatology and Endocrinology, Medical University of Innsbruck, Innsbruck, Austria

**Keywords:** artificial intelligence, bone mineral density, glucocorticoids, imaging, immunosuppression, kidney transplantation

## Abstract

Mineral and bone disease (MBD) is a frequent complication after solid organ transplantation. The rapid decline in bone mineral density (BMD) during the first year after kidney transplantation reflects complex interactions between immunosuppressive therapy, metabolic factors and pre-existing chronic kidney disease (CKD)-MBD.

This narrative review summarizes the magnitude and time course of BMD loss after kidney transplantation, the underlying metabolic and pharmacologic mechanisms, and associated fracture risk. Diagnostic strategies including dual-energy X-ray absorptiometry (DXA), quantitative computed tomography (QCT), high-resolution peripheral QCT(HR-pQCT), finite element analysis (FEA), and biochemical markers are reviewed alongside preventive and therapeutic approaches.

Recent data highlight early deterioration of cortical bone structure, persistent hyperparathyroidism, and the limited predictive value of DXA alone. Integration of advanced imaging and bone-turnover markers as well as markers of mineral metabolism could improve risk stratification.

Emerging artificial intelligence-based modeling and HR-pQCT-guided phenotyping may enable individualized prevention strategies in kidney transplant recipients (KTRs).

Although advances in immunosuppressive therapy have improved graft and patient survival, chronic metabolic consequences, including osteoporosis and osteomalacia, have become increasingly important.^1^^,^^2^ The rapid BMD decline during the first post-transplant year reflects interactions between therapy-related and patient-specific factors, including exposure to high-dose corticosteroids and/or calcineurin inhibitors, immobilization, persistent hyperparathyroidism, and vitamin D deficiency.^3^^,^^4^ Persistent elevation of fibroblast growth factor-23 (FGF23) may additionally drive renal phosphate wasting and transient hypophosphatemia, impairing mineralization and contributing to early cortical deterioration.^5^ Consequently, fracture risk is high among solid-organ transplant recipients, with vertebral and nonvertebral fractures substantially increasing morbidity and potentially compromising graft outcomes.^6^, ^7^, ^8^, ^9^, ^10^, ^11^

Although DXA remains the standard method for assessing bone mineral content, its pixel resolution of approximately 1 × 1 mm and 2-dimensional projection limit detection of microarchitectural deterioration and cortical porosity.^12^ Although QCT resolves the problem of the separation of trabecular bone from cortical bone, only HR-pQCT provides information on cortical thickness and porosity that are important parameters the biomechanical modeling of bone strength that underlies estimation of failure loads by FEA.^13^^,^^14^

This review summarizes current evidence on early BMD loss, provides an overview of available diagnostic tools including HR-pQCT and FEA, and outlines preventive strategies tailored to metabolic risk factors and immunosuppressive therapy. In addition, it integrates emerging insights into CKD-MBD legacy effects, microarchitectural imaging, and individualized fracture risk assessment in KTRs.

### Bone Mineral Density Loss in the First Post-Transplant Year

#### Magnitude and Time Course of BMD Loss

BMD declines rapidly during the first post-transplant year, particularly in trabecular-rich sites such as the spine, femoral neck, and distal radius.^15^ However, early post-transplant BMD trajectories are heterogenous, although aggregate data suggest mean losses of 4% to 10% at the lumbar spine and 3% to 8% at the femoral neck within 6 to 12 months,^6^^,^^16^ individual responses vary considerably. In a contemporary cohort of *de novo* KTRs treated under a steroid-minimization protocol, only the ultradistal radius showed a significant overall decline, whereas femoral neck areal BMD (aBMD) decreased, remained stable, or increased in approximately equal proportions of recipients.^17^

The steepest decline occurs during the initial months of highest glucocorticoid exposure.^7^^,^^18^ Contemporaneous steroid-sparing regimens have substantially reduced early BMD loss compared with historical protocols^9^ (see Figure 1 for an overview).

**Figure 1:**
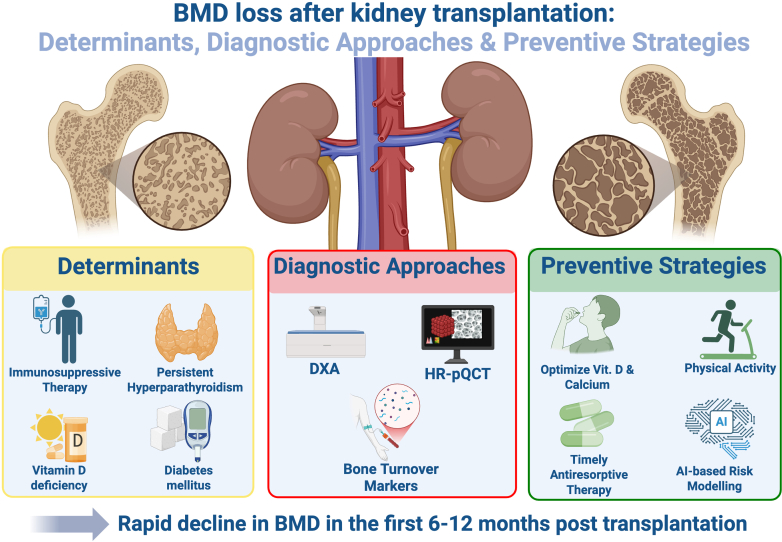
Overview of bone mineral density loss after kidney transplantation: determinants, diagnostic approaches, and preventive strategies. This overview summarizes the major determinants, diagnostic modalities and preventive interventions related to bone mineral density (BMD) loss in kidney transplant recipients. Early post-transplant bone deterioration is driven by immunosuppressive therapy, persistent hyperparathyroidism, vitamin D deficiency and diabetes mellitus. Diagnostic evaluation combines dual-energy X-ray absorptiometry (DXA), high-resolution peripheral quantitative computed tomography (HR-pQCT) and biochemical bone turnover markers to characterize trabecular and cortical changes. Preventive measures include optimization of vitamin D and calcium status, early initiation of antiresorptive therapy in high-risk individuals, structured physical activity, and emerging artificial intelligence (AI)-based risk modeling. Collectively, these elements highlight the rapid decline in BMD occurring within the first 6 to 12 months after transplantation and emphasize the importance of timely assessment and intervention.

### Contributing Clinical Factors

#### Pre-Existing CKD-MBD

Most KTRs present with pre-existing CKD-MBD. Persistent hyperparathyroidism is a common finding in KTRs and an important cause of increased bone remodeling, hypercalcemia, and hypophosphatemia.^1^^,^^2^^,^^19^ These metabolic derangements may accelerate bone loss, and as such contribute to the excessive fracture in incident KTRs.^20^ In advanced stages of CKD, the concentration of circulating intact FGF23 may rise markedly as an adaptive response to maintain phosphate balance, but chronically high intact FGF23 can become a key driver of CKD-MBD. Circulating intact FGF23 acts via FGFR-Klotho to internalize the sodium-phosphate cotransporters, NaPi-2a and NaPi-2c, from the apical plasma membrane of proximal tubular epithelial cells, thereby increasing urinary phosphate excretion.^21^ At the same time, intact FGF23 suppresses renal 1α-hydroxylase (encoded by *CYP27B1*) and stimulates 24-hydroxylase (encoded by *CYP24A1*), leading to reduced calcitriol levels and contributing to secondary hyperparathyroidism. When patients with advanced CKD and markedly elevated pretransplant intact FGF23 receive a kidney graft, the restored filtration capacity unmasks the hormone’s phosphaturic effect, resulting in pronounced urinary phosphate wasting and early post-transplant hypophosphatemia. Persistently high intact FGF23 plasma concentrations in the early post-transplant period have been associated with significant BMD loss at the lumbar spine and hip, independent of other metabolic factors. Thus, elevated intact FGF23 not only counteracts or attenuates hyperphosphatemia in patients with advanced CKD, but also causes calcitriol deficiency and predisposes to accelerated BMD deterioration once glomerular filtration and other kidney functions are restored by transplantation.^22^ Observational studies in KTRs showed that persistently elevated intact FGF23 in the early post-transplant period is strongly associated with hypophosphatemia and loss of BMD, particularly at the lumbar spine and hip, independent of other factors.^23^^,^^24^

#### Mobility and Nutrition

Reduced physical activity, postoperative immobilization, and poor nutrition, particularly insufficient dietary protein and calcium intake, exacerbate post-transplant bone loss, whereas early exercise and training programmes mitigated this decline.^4^

#### Metabolic and Pharmacologic Determinants

Post-transplant bone loss results from combined pharmacologic and metabolic disturbances, including glucocorticoid toxicity, the skeletal effects of calcineurin inhibitors and mechanistic target of rapamycin inhibitor, and persistent CKD-MBD abnormalities. Inflammation, diabetes and malnutrition further modulate skeletal turnover.

#### Glucocorticoid-Induced Loss of Bone Density

Glucocorticoids remain the most influential clinical determinant of early BMD decline. They inhibit osteoblast differentiation, induce osteocyte apoptosis, and prolong osteoclast survival, resulting in net resorption during the first 3 to 6 months after kidney transplantation.^7^ Clinically, decreases of 4% to 9% at the lumbar spine and 5% to 8% at the hip are observed during the period of highest cumulative exposure,^7^^,^^25^^,^^26^ and the severity of bone loss correlates most strongly with dose intensity and tapering speed.^27^, ^28^, ^29^

#### Calcineurin and Mechanistic Target of Rapamycin Inhibitors

Data on the skeletal impact of nonglucocorticoid immunosuppressive drugs are scarce and inconsistent. Experimental and clinical evidence suggested that calcineurin inhibitors may have adverse effects on bone metabolism, though the magnitude and clinical relevance of these effects in KTRs remain uncertain.^30^ Similarly, mechanistic target of rapamycin inhibitors have been suggested to interfere with osteoblast function, but human data are limited.^31^ Although these findings call for additional evidence, they underscore the need for individualized immunosuppressive regimens balancing benefits and (skeletal) risks.^7^^,^^32^

#### Legacy Effects of CKD-MBD

The metabolic abnormalities characteristic of CKD-MBD frequently persist after transplantation and substantially hamper early skeletal recovery. Persistent hyperparathyroidism drives bone remodeling activity, which most prominently manifests by deteroriation of cortical microarchitecture.^25^^,^^30^ Early post-transplant hypophosphatemia, primarily driven by elevated FGF23 and parathyroid hormone (PTH), may impair mineralization and delay normalization of BMD.^7^ Vitamin D deficiency often persists after kidney transplantation and may be involved in the pathogenesis of both persistent hyperparathyroidism and mineralization defects.^25^

Collectively, these legacy abnormalities contribute significantly to the increased fracture risk, especially in the early post-transplant period.

#### Diabetes and Metabolic Comorbidities

Both pre-existing type 2 diabetes and new-onset diabetes after transplant are associated with increased fracture risk in KTRs.^7^^,^^33^ Hyperglycemia impairs osteoblast function and promotes accumulation of advanced glycation end-products that reduce bone material quality independent of BMD.^34^

#### Additional Modifiers: Inflammation, Oxidative Stress, Sarcopenia, Malnutrition

Chronic inflammation and oxidative stress, common in CKD and early after transplantation, promote osteoclast activation and impair osteoblast survival, limiting post-transplant skeletal recovery.^7^^,^^27^ Inflammatory cytokines such as interleukin-6 and tumor necrosis factor -α accelerate bone resorption during periods of acute rejection or infection.^35^, ^36^, ^37^ Gut dysbiosis and impaired intestinal barrier function may further amplify systemic inflammation and contribute to CKD-MBD pathophysiology, including post-transplant bone loss.^38^ Sarcopenia reduces mechanical loading and malnutrition restricts key substrates, such as protein, calcium and vitamin D, collectively increasing fracture risk in transplant recipients.^25^^,^^39^ Immunosuppressive therapy may additionally influence nutritional status and lean mass, compounding musculoskeletal vulnerability.^39^ Neuro-psycho-cognitive medications may represent an additional contributor to fracture risk in KTRs. Antidepressants, antipsychotics, benzodiazepines, and certain antiepileptic drugs are associated with increased fall risk, impaired balance, and in some cases direct effects on bone metabolism.^40^^,^^41^ Given the high prevalence of sleep disorders, anxiety, and depression in transplant populations, these medications may represent an underrecognized but potentially modifiable determinant of fracture risk.

### Fracture Risk and Clinical Outcomes

#### Epidemiology of Fractures After Transplantation

Fractures remain a frequent complication after kidney transplantation. Recent cohorts reported fracture incidence rates ranging from 7 to 17 per 1000 patient-years for all fractures and approximately 1.5 per 1000 patient-years for hip fractures, with the highest rates observed during the first post-transplant year.^6^^,^^33^ Older cohorts demonstrated a several-fold increase compared with the general population, particularly among female recipients, whose relative fracture risk was reported up to 20-fold higher than age-matched controls.^42^ Notably, registry-based data spanning 2 decades suggested that fracture risk after kidney transplantation has remained largely unchanged over time and continues to be inadequately addressed in clinical practice.^43^

#### Predictive Factors for Fracture

Clinical determinants of incident fractures include older age, biological sex, diabetes mellitus, dialysis vintage, and cumulative glucocorticoid exposure.^33^ Persistent hyperparathyroidism, vitamin D deficiency, and impaired graft function can further compromise skeletal integrity.^44^ Among modifiable factors, early glucocorticoid tapering and steroid-minimization regimens significantly mitigate early bone loss and fracture risk.^9^

The fracture risk assessment tool (FRAX) provides a structured approach to fracture risk estimation in KTRs. In a cohort of 458 KTRs, the observed 10-year risk for major osteoporotic fractures was 6.3% (95% confidence interval: 3.4–9.2%), with FRAX-predicted risk of 5.0% with BMD and 5.6% without BMD.^45^ The addition of BMD did not substantially improve prediction accuracy, and hazard ratios for fracture were essentially similar between models,^45^ suggesting that CKD-related bone alterations may limit the incremental value of DXA-derived BMD for fracture risk stratification in this population. FRAXplus is an enhanced version of the original FRAX tool allowing more refined input of glucocorticoid exposure (dose and duration) and falls.^46^ FRAXplus incorporates transplant-relevant variables and may therefore refine fracture prediction in KTRs, though direct comparative evidence remains limited.^47^, ^48^, ^49^

#### Clinical and Functional Consequences

Fractures substantially increase morbidity and mortality. Hip fractures double mortality risk, whereas vertebral and limb fractures also raise the likelihood of graft loss and death.^50^ Moreover, patients sustaining a fracture had an approximately 30% higher risk of subsequent graft failure.^50^ Functional outcomes are equally important; vertebral fractures lead to chronic pain, spinal deformity, and reduced mobility, whereas hip fractures frequently result in long-term disability and loss of independence. These events markedly reduce quality of life, as confirmed by contemporary analyses of solid-organ transplant populations.^51^

Quantitative assessment of fracture risk and preventing fractures is therefore essential to preserve not only graft survival but also physical function and life expectancy in KTRs.

### Diagnostic Approach

Assessment of skeletal health after transplantation requires integration of clinical risk factors, imaging and biochemical markers. Fracture risk tools such as FRAX or FRAXplus may complement imaging (Figure 2 for an overview).

**Figure 2:**
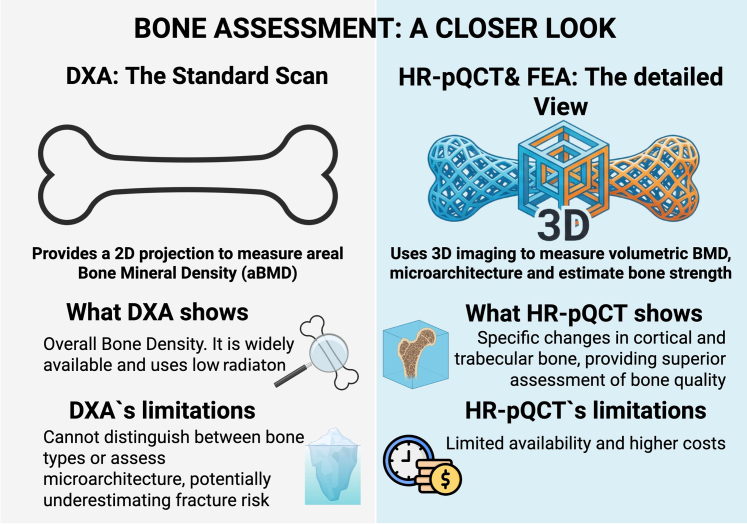
Bone assessment methods: DXA versus HR-pQCT and FEA. This figure provides a comparative overview of commonly used bone assessment techniques. Dual-energy X-ray absorptiometry (DXA) offers a 2-dimensional projection to measure areal bone mineral density (aBMD) and is widely available with low radiation exposure. However, DXA cannot differentiate cortical from trabecular bone or characterize bone microarchitecture, which may lead to underestimation of fracture risk. High-resolution peripheral quantitative computed tomography (HR-pQCT), combined with finite element analysis (FEA), provides 3-dimensional imaging to evaluate volumetric bone mineral density (vBMD), cortical and trabecular microarchitecture and to estimate bone strength. HR-pQCT allows more detailed assessment of bone quality but is limited by higher costs and reduced availability.

#### DXA

DXA is the standard technique for assessing BMD in organ transplant recipients. It operates by measuring the differential attenuation of X-rays at 2 energies, providing aBMD at the lumbar spine, hip, and forearm. The strengths of this investigation include wide availability, low radiation exposure, and robust fracture risk prediction, particularly at the hip and spine. However, DXA cannot distinguish between trabecular and cortical bone, nor does it assess bone microarchitecture or quality, which are often altered in transplant recipients because of glucocorticoid exposure and persistent or *de novo* metabolic disturbances. Additionally, DXA may underestimate fracture risk in patients with preserved BMD but abnormal bone quality.^6^^,^^7^^,^^52^ The trabecular bone score can be derived from lumbar spine DXA images and serves as a proxy for trabecular microarchitecture. Trabecular bone score adds prognostic information independent of aBMD because a lower trabecular bone score was associated with an approximately 64% higher fracture risk per SD decrease in KTRs.^53^ Trabecular bone score may identify skeletal deterioration not captured by aBMD alone, underscoring its utility as a complementary DXA-derived parameter.^53^^,^^54^

#### HR-pQCT

HR-pQCT utilizes 3-dimensional imaging to quantify volumetric BMD of cortical and trabecular bone separately. In addition, its high spatial resolution allows assessment of microarchitectural parameters including trabecular number, thickness, separation, cortical thickness, and porosity. Compared with DXA, HR-pQCT provides detailed characterization of cortical and trabecular microarchitecture and has demonstrated strong associations with fracture risk in the general population. However, data specifically in KTRs remain limited, and most available evidence derives from small cohorts or extrapolation from CKD and other transplant populations. Key limitations include restricted availability, higher costs, and the absence of transplant-specific fracture risk thresholds. Age- and sex-specific reference values have recently been published, improving interpretability of HR-pQCT measurements.^55^ Although HR-pQCT-derived volumetric BMD and microarchitectural parameters predicted fracture risk in nontransplant populations,^56^ robust data in KTRs are still scarce.

#### FEA

FEA can be derived from HR-pQCT images. FEA estimates failure load, a surrogate for biomechanical competence and bone strength. FEA models the bone’s response to mechanical stress, providing a direct measure of bone quality beyond BMD alone. In transplant recipients, FEA-derived failure load correlates with microarchitectural deterioration and may improve fracture risk stratification, though clinical validation is ongoing.^57^

#### Biochemical and Bone Turnover Markers

Biochemical markers of mineral metabolism and bone turnover include, among others, serum calcium (total and ionized), phosphate, parathyroid hormone, vitamin D metabolites, intact FGF23, bone formation markers such as total and bone-specific alkaline phosphatase, and total and intact procollagen type 1 N-terminal propeptide, as well as bone resorption markers including C-terminal telopeptide of type I collagen and Tartrate-resistant acid phosphatase 5b (see Table 1). These change dynamically after kidney transplantation,^19^^,^^58^ reflecting the shift from the pretransplant CKD-MBD milieu toward post-transplant alterations in mineral metabolism.^9^ Low plasma concentrations of 25-hydroxyvitamin D remain common, detectable in up to 80% of recipients within 3 months after transplantation.^9^ Vitamin D deficiency has been associated with secondary hyperparathyroidism^59^^,^^60^ and may contribute to impaired mineralization; however, its direct causal role in cortical bone loss in the early post-transplant setting remains to be fully elucidated.^60^^,^^61^ Because several bone turnover markers, including C-terminal telopeptide of type I collagen, are partly renally cleared, their interpretation in CKD and early after transplantation is challenging.^62^^,^^63^ Recent work therefore highlighted nonkidney-cleared markers such as intact procollagen type 1 N-terminal propeptide, bone-specific alkaline phosphatase and tartrate-resistant acid phosphatase 5b as more reliable indicators,^64^^,^^65^ with a recent European Renal Osteodystrophy consensus statement further defining their role.^66^

**Table 1:** Overview of key biochemical markers used to assess bone turnover after kidney transplantation. This table summarizes commonly used biochemical markers relevant to post-transplant bone assessment, including their physiological role, typical post-transplant patterns, clinical utility, and important limitations. Nonkidney-cleared markers such as intact P1NP, BALP, and TRAP5b provide more reliable information on bone turnover in the early post-transplant period, whereas renally cleared markers such as CTx may be misleading until graft function stabilizes

Marker	Physiological meaning	Changes after transplantation	Clinical utility	Limitations
PTH	Regulates calcium/phosphate homeostasis; reflects bone turnover	Declines by ∼ 30–60% during the first year, but persistent elevations are common	Key indicator of high-turnover bone disease; persistent hyperparathyroidism contributes to cortical loss	Low levels rarely indicate true low-turnover states; influenced by vitamin D & graft function
25-OH Vitamin D	Reflects vitamin D stores; influences PTH and mineralization	Deficiency frequent (> 80% early post-transplant)	Low levels associated with cortical thinning, impaired mineralization	Affected by sunlight, nutrition; cut-offs not transplant-specific
BALP (Bone-specific alkaline phosphatase)	Marker of osteoblast activity and bone formation	Typically normalizes with improving mineral metabolism	Useful non renally-cleared marker of bone formation	May be elevated in liver/biliary disease; modest biological variability
Intact P1NP	Marker of type I collagen formation; bone formation	Dynamic changes during early post-transplant period	Preferred formation marker (not renally cleared); helps distinguish high vs. low turnover	Mild diurnal variation; assay variability across platforms
TRAP5b	Osteoclast number and bone resorption	Provides stable measurement independent of kidney function	Reliable non–kidney-cleared resorption marker	Limited availability in some centers
CTx (C-terminal telopeptide of type I collagen)	Osteoclast-mediated bone resorption	May fall rapidly post-transplant; levels affected by GFR recovery	Can track changes in turnover when GFR is stable	Renally cleared **→** overestimates turnover in CKD or early post-transplant

CKD, *chronic kidney disease*; GFR, glomerular filtration r*ate;* P1NP, procollagen type 1 N-terminal propeptide; TRAP5b, tartrate-resistant acid phosphatase 5b.

Elevated PTH levels represent the main biochemical abnormality associated with poor skeletal health post-transplant. PTH typically declines rapidly within the first 3 to 6 months after transplantation, followed by a more gradual decline that plateaus at approximately 1 year.^67^ Despite this initial improvement, many patients exhibit persistent hyperparathyroidism, with up to 50% maintaining inappropriately elevated PTH levels years after successful transplantation.^67^^,^^68^ These persistently high levels may sustain high bone turnover and promote cortical bone loss.^6^^,^^20^

Integrating biochemical markers with imaging enhances risk stratification, such as PTH, must be interpreted in aggregate with calcium and phosphate and trends are more informative than single time point values. Correction of PTH, mineral metabolism disturbances, and nutritional vitamin D stores, preferentially guided by nonkidney-cleared bone turnover markers are key in improving post-transplant bone health and preventing fractures.^58^

### Bone Biopsy and Histomorphometry

Bone biopsy with histomorphometric analysis provides direct information on bone turnover, mineralization, and bone volume that cannot be obtained noninvasively, and can identify the predominant skeletal pathology,^69^ such as high-turnover disease, adynamic bone, or osteomalacia,^70^ with direct therapeutic implications. However, bone biopsy is invasive, available only in specialized centers, and its role in KTRs is therefore best reserved for cases where noninvasive assessment is inconclusive and the underlying bone pathology is likely to change clinical management.^25^

### Preventive and Therapeutic Strategies

Prevention of post-transplant bone loss should begin before kidney transplantation and continue through the early postoperative phase.^71^ The approach combines general measures (calcium, vitamin D optimization, and weight-bearing activity) and pharmacologic strategies tailored to bone turnover status and graft function.^72^, ^73^, ^74^ Although data on how to best reduce fracture risk remain limited, consistent evidence supports early correction of secondary hyperparathyroidism and timely initiation of antiresorptive therapy in high-risk recipients.^75^^,^^76^

#### Nonpharmacologic Management

Early mobilization and physical therapy are recommended to mitigate bone loss, as weight-bearing exercise stimulates bone formation and reduces fracture risk.^72^^,^^77^ Nutritional interventions including adequate calcium, protein, and vitamin D supplementation are essential, as low plasma vitamin D concentrations and secondary hyperparathyroidism are common and contribute to bone loss; oral calcium and cholecalciferol supplementation attenuates BMD loss and lowers PTH after kidney transplantation.^73^^,^^75^^,^^78^ Supplementation is typically recommended when low 25-hydroxyvitamin D plasma concentration is documented, although many centers apply a prophylactic strategy in the early post-transplant period because of the high prevalence of vitamin D deficiency.^59^^,^^60^ Evidence does not clearly favor a strictly prophylactic versus reactive approach,^61^ highlighting the need for individualized vitamin D management based on baseline levels and biochemical monitoring.^79^ Recent European Renal Osteodystrophy consensus recommendations further emphasize the importance of maintaining adequate nutritional vitamin D and calcium intake in CKD and post-transplant populations, providing updated guidance on optimal targets and clinical monitoring.^60^^,^^80^

#### Pharmacologic Prevention

Pharmacologic prevention plays a central role in mitigating the rapid early decline in BMD after transplantation. The principal pharmacologic classes used for prevention and treatment of post-transplant bone loss and their mechanisms of action are summarized in Figure 3. Although long-term fracture data remain limited, intervention beyond the immediate post-transplant period (i.e., after the first 3 months) is justified given the persistently elevated fracture risk. Preventive strategies should therefore be applied proactively in patients with high or very high fracture risk as predicted by FRAX, particularly in those with previous low-trauma fractures, tertiary hyperparathyroidism, sustained glucocorticoid exposure, or early post-transplant BMD loss.^81^, ^82^, ^83^

**Figure 3:**
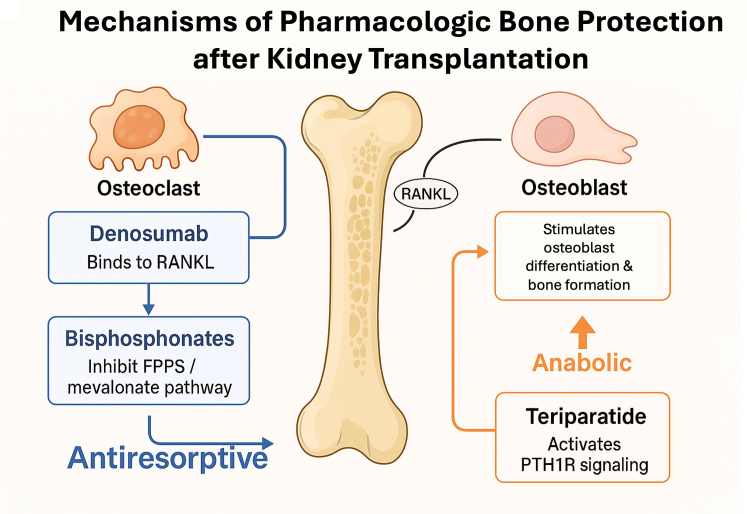
Mechanisms of pharmacologic bone protection after kidney transplantation. Schematic representation of the principal mechanisms of antiresorptive and anabolic agents acting on bone cells. Bisphosphonates inhibit FPPS in the mevalonate pathway within osteoclasts, leading to reduced bone resorption and osteoclast apoptosis. Denosumab, a monoclonal antibody against RANKL, prevents RANKL from binding to its receptor on osteoclast precursors, thereby inhibiting osteoclast differentiation and activity. Teriparatide, a recombinant PTH 1-34 analogue, activates PTH1R signaling on osteoblasts, stimulating bone formation and remodeling. The balance between antiresorptive and anabolic effects determines overall bone mineral density preservation after transplantation. The arrow from denosumab to bisphosphonates indicates the recommended sequential strategy: upon denosumab discontinuation, a bisphosphonate should be promptly initiated to prevent rebound-associated bone loss and vertebral fractures. FPPS, farnesyl pyrophosphate synthase; PTH, parathyroid hormone; RANKL, receptor activator of nuclear factor-κB ligand.

If antiresorptive therapy is not or cannot be prophylactically initiated during the early post-transplant period, treatment decisions should rely on an integrated fracture-risk assessment also considering potential contraindications against antiresorptive therapy rather than BMD alone.^71^ Relevant determinants include previous fractures, femoral neck BMD, glucocorticoid exposure, age, diabetes, smoking, comorbidities and persistent hyperparathyroidism, supported by tools such as FRAX or FRAXplus. Current guidelines recommend considering antiosteoporotic therapy based on absolute fracture risk rather than fixed T-score cutoffs, for example when the predicted 5-year risk of a major osteoporotic fracture exceeds 10%, or in the presence of long-term glucocorticoid therapy (e.g., oral prednisone ≥7.5 mg daily).^83^, ^84^, ^85^ Future transplant-specific refinements of FRAX may further improve individualized stratification.^84^

Table 2^86^^,^^87^ summarizes granular clinical trial data for bisphosphonates and denosumab.

**Table 2:** An overview over key studies assessing the efficacy and safety of pharmacological interventions in KTRs

Study (Ref)	Design & population	Intervention	Comparator	Follow up (Mo)	Skeletal outcomes	Safety notes
Bonani *et al.*^1^^,^^8^	RCT; de novo KTR	Denosumab 60 mg s.c. every 6 months, early post-Tx	Placebo	12	↑ BMD at LS and TH; prevented early bone loss	Monitor Ca/P; hypocalcemia risk if low Vit D
Bonani *et al.*^1^^,^^81^	HR-pQCT sub-study; de novo KTR	Denosumab (as above)	Untreated	12	Improved cortical metrics and FEA-derived strength	No major AEs; microarchitectural benefit
Marques *et al.*^86^	RCT; de novo KTR	Zoledronic acid 5 mg i.v. early post-Tx	Placebo	12	Preserved BMD vs. placebo	Avoid if eGFR < 30 ml/min per 1.73 m^2^; acute-phase reaction
Sayed *et al.*^87^	Active-comparator RCT; KTR with low BMD	Denosumab 60 mg s.c. every 6 mos	Alendronate	12	Both ↑ BMD; denosumab noninferior / slightly superior	Safe in reduced eGFR; monitor Ca/P

AEs, adverse events; BMD, bone mineral density; eGFR, estimated glomerular filtration rate; FEA, finite element analysis; HR-pQCT, high-resolution peripheral quantitative computed tomography; KTR, kidney transplant recipient; LS, lumbar spine; RCT, randomized controlled trial; TH, total hip; Tx, transplantation.

##### Bisphosphonates

Bisphosphonates are potent antiresorptive agents that exhibit a high affinity for hydroxyapatite in bone. This class of drugs is the best studied for prevention of BMD loss after organ transplantation. They consistently increase BMD at the lumbar spine and femoral neck in kidney and other solid organ transplant recipients, with meta-analyses showing a reduction in fracture risk and bone pain, although the certainty regarding fracture reduction remains low and data on long-term outcomes are limited.^76^^,^^88^, ^89^, ^90^ Concerns traditionally centered on the development of “adynamic bone disease” have been increasingly questioned, as low bone turnover is an expected pharmacologic effect of antiresorptive therapy and does not necessarily represent a pathological state.^91^ Bisphosphonates should still be used with caution in patients with significantly reduced kidney function because of renal clearance and potential accumulation,^72^^,^^86^^,^^90^ although recent European Renal Osteodystrophy guidance supports a more liberal use even in advanced CKD (G4-5D) under appropriate clinical monitoring.^92^

In patients with very high fracture risk and concerns about adherence or impaired oral absorption bisphosphonates can and should be administered i.v. Zolendronic acid is effective in reducing fractur risk. Accordingly, an open label study on the effect of zolendronic acid in KTRs confirmed its positive effect on bone quality as assessed by HRpQCT, however the control group did not show significant bone loss either, when a contemporaneous immunosuppressive regimen was used.^86^

##### Denosumab

Unlike bisphosphonates, denosumab does not incorporate into bone and its antiresorptive effects are rapidly reversible upon discontinuation,^81^^,^^82^ which typically causes a rebound effect when denosumab is stopped, which requires specific risk mitigation staregies.^93^

Randomized trials and cohort studies in kidney and other solid organ transplant recipients demonstrate that denosumab significantly increases BMD at the lumbar spine, hip, and radius, with efficacy comparable to alendronate; moreover, short-term safety over 12 months appears favorable.^87^^,^^94^ However, fracture-end point data in KTRs remain limited, and robust evidence for fracture-risk reduction is still lacking, similar to gaps seen with bisphosphonates.^94^

Withdrawal of denosumab is consistently associated with a rebound phenomenon, characterized by rapid increases in bone turnover markers and accelerated BMD loss,^95^^,^^96^ which may lead to vertebral fractures, especially after longer denosumab therapy.^97^ This is clinically relevant in KTRs, who often have additional risk factors for skeletal fragility.^48^ If denosumab discontinuation becomes necessary, for example, because of hypocalcemia or intolerance, initiation of a bisphosphonate before the next missed dose is strongly recommended to prevent rebound-associated bone loss.^95^^,^^96^^,^^98^

Because of this rebound risk, denosumab should ideally not be discontinued once initiated, unless an alternative antiresorptive agent is promptly introduced.^95^^,^^99^

##### Teriparatide

Teriparatide^7^ ^(pp1–34)^ is an osteoanabolic agent that stimulates bone formation through intermittent activation of the PTH1 receptor (Figure 2).^100^, ^101^, ^102^ The previous treatment limit of 24 months has been removed in the United States, allowing extended use in patients who remain at high fracture risk, although long-term safety data beyond 2 years remain limited.^103^

However, evidence in KTRs is scarce. In a randomized double-blind trial, daily subcutaneous teriparatide stabilized femoral neck BMD but did not significantly improve lumbar spine or radial BMD compared with placebo.^104^ Teriparatide also did not enhance bone turnover or mineralization in this setting. A more recent retrospective observational study in KTRs reported real-world use of teriparatide with some improvements in BMD, though the study was small and uncontrolled.^105^ Nevertheless, it may have a role in selected post-transplant patients with severe hypocalcemia and low PTH levels. Case series suggest that teriparatide can normalize serum calcium in parathyroidectomized recipients with refractory hypocalcemia.^106^

##### Abaloparatide

Abaloparatide, a synthetic analogue of parathyroid hormone-related protein (PTHrP 1–34), is an osteoanabolic agent with bone-forming effects similar to teriparatide but more selective activation of the PTH1 receptor,^107^ which may result in lower stimulation of bone resorption.^108^ Clinical trials in postmenopausal osteoporosis demonstrate robust increases in BMD and fracture-risk reduction.^48^^,^^109^ However, data in KTRs or advanced CKD populations are currently lacking,^110^ and its role in transplant-related bone disease therefore remains uncertain. Abaloparatide is currently approved for the treatment of postmenopausal osteoporosis but not for CKD-related bone disease. Longer-acting PTH analogues such as palopegteriparatide are currently being investigated primarily for hypoparathyroidism, and their potential role in transplant-related bone disease remains unknown.^111^^,^^112^

##### Romosozumab

Romosozumab, a monoclonal antibody targeting sclerostin, exerts a dual effect by simultaneously stimulating bone formation and reducing bone resorption, with proven efficacy in postmenopausal osteoporosis.^113^ Evidence in KTRs remains limited to small retrospective case series demonstrating BMD improvements in patients with severe post-transplant osteoporosis.^113^ Romosozumab should be used with caution in patients with elevated cardiovascular risk, as clinical trial data and Mendelian randomization analyses have raised concerns about adverse cardiovascular events with sclerostin inhibition.^114^^,^^115^ It may therefore be considered in carefully selected recipients with severe osteoporosis who have exhausted or are intolerant of standard antiresorptive options, but robust controlled trial data are currently lacking.

### Summary and Outlook

Studies indicate that early initiation of pharmacologic bone-active therapy after transplantation, either as risk-based primary prevention in patients at high fracture risk or as treatment of established osteoporosis, is associated with greater preservation of bone mass and reduced early fracture risk, reflecting the pronounced bone loss during the first 6 to 12 months after transplantation.^72^^,^^73^^,^^76^^,^^88^ Delaying therapy until substantial BMD loss becomes evident may therefore miss a window of maximal therapeutic benefit.

However, an alternative strategy deserves consideration: measuring BMD before transplantation and repeating DXA at 3 or 6 months post-transplant to detect early deterioration. This approach would allow for targeted initiation of antiresorptive therapy if BMD declines or switching to i.v. bisphosphonates or to osteoanabolic treatment, even if values remain in the nonosteoporotic range.^72^ Such a framework requires repeated fracture-risk assessment but may help identify individuals at risk while avoiding unnecessary prophylaxis. Importantly, this approach also necessitates an understanding of the precision error of the measurement system and the least significant change that can be reliably detected, as these determine whether observed differences in serial BMD values reflect true biological change rather than measurement variability.^116^^,^^117^ In addition, monitoring nonkidney-cleared bone turnover markers, such as intact procollagen type 1 N-terminal propeptide, bone-specific alkaline phosphatase, and tartrate-resistant acid phosphatase 5b, may provide an early biochemical signal of turnover changes that precede measurable declines in BMD, as suggested by recent data.^19^

At the same time, prophylactic antiresorptive therapy may be criticized, as its effects on hard clinical outcomes, particularly fracture risk reduction, remain insufficiently established in transplant populations.^71^ This evidence gap underscores the need for adequately powered randomized trials comparing prophylactic versus BMD-triggered treatment strategies. Future studies should therefore aim to clarify the optimal timing of therapy, incorporate early and repeated imaging, and identify subgroups that benefit most from either approach.

### Emerging Therapies

Artificial intelligence approaches have been proposed to improve fracture risk assessment by integrating clinical, biochemical and imaging variables. However, evidence in KTRs remains very limited, and current applications are largely exploratory.^7^^,^^11^^,^^118^ At present, artificial intelligence (AI)-based approaches are not validated for routine clinical use in transplant populations.

### Legacy Management of CKD-MBD in the Post-Transplant Setting

Residual CKD-MBD abnormalities inherited from the dialysis period (“legacy” CKD-MBD) frequently persist after kidney transplantation and substantially influence early bone turnover and long-term skeletal health. Many recipients enter the post-transplant period with sustained persistent hyperparathyroidism, altered set-points for calcium-PTH regulation, and structural bone changes that do not immediately normalize despite improved kidney function.^7^^,^^74^^,^^119^^,^^120^

Persistent hyperparathyroidism remains a major contributor to high-turnover bone disease and cortical deterioration in the first post-transplant year.^120^^,^^121^ In addition to persistent PTH elevation, some studies have described the concept of “tertiary FGF23 elevation” referring to sustained or recurrent increases in FGF23 despite improved graft function. Such elevations may contribute to ongoing renal phosphate wasting and impaired skeletal mineralization, thereby perpetuating post-transplant mineral metabolism disturbances.^5^^,^^122^

In this context, calcimimetic therapy (e.g., cinacalcet) may be continued or reinitiated after transplantation to reduce PTH levels and correct hypercalcemia and hypophosphatemia.^123^^,^^124^ A pivotal randomized placebo-controlled trial demonstrated clear biochemical efficacy of cinacalcet in correcting hypercalcemia and elevated PTH; however, no significant benefit on femoral neck BMD was observed, and a clear skeletal advantage was not established.^125^ Thus, cinacalcet should be considered primarily as a biochemical intervention rather than a bone-protective therapy per se. Evidence suggests that it may reduce the need for parathyroidectomy in selected patients.^125^^,^^126^

Parathyroidectomy remains an effective strategy for correcting severe biochemical disturbances in KTRs with persistent hypercalcemic hyperparathyroidism unresponsive to medical therapy.^127^ Compared with cinacalcet, available comparative data suggest a more favorable signal for parathyroidectomy with respect to normalization of calcium and PTH, and at least some evidence points to skeletal benefits, though fracture end point data remain limited.^125^^,^^126^ A recent transplant-oriented review by Cianciolo *et al.* further highlights the asymmetry between biochemical and skeletal outcomes when comparing these 2 approaches, and provides a useful framework for individualizing management.^128^

### Future Perspectives

The next decade will likely refine transplant bone diagnostics through incremental advances in imaging, computational analysis, and individualized management strategies. HR-pQCT offers detailed assessment of trabecular and cortical microarchitecture, and AI-assisted FEA modeling may enhance fracture-risk prediction beyond traditional BMD thresholds. However, large multicenter studies are required to validate these approaches, assess cost-effectiveness, and establish standardized cut-offs.^7^^,^^129^

Although HR-pQCT is increasingly explored as a complementary or potential alternative to DXA in transplant populations, its routine clinical application will primarily depend on broader availability and on robust clinical studies demonstrating superiority over DXA in predicting fracture risk or monitoring treatment response.^7^^,^^129^

AI-based segmentation tools and integration into picture archiving and communication systems may support automated and reproducible assessment of bone microstructure, though widespread adoption requires validated reference standards and standardized reporting frameworks.^7^

Personalized approaches integrating imaging, biochemical markers and clinical phenotyping may improve fracture-risk stratification in transplant recipients.^7^^,^^71^^,^^73^ Current Kidney Disease: Improving Global Outcomes recommendations already emphasize comprehensive evaluation of bone health in transplant candidates and recipients and support the need for tailored interventions.^71^

Future research priorities include longitudinal comparisons of FRAXplus with or without DXA and HR-pQCT for fracture prediction and monitoring of bone recovery,^7^^,^^129^ development of multicenter normative datasets to improve interpretability, and standardized reporting of imaging and biomarker results (see Table 3 for an overview).^7^^,^^71^

**Table 3:** Key unanswered questions and areas requiring additional evidence. This table outlines major unresolved questions in the management of bone disease after kidney transplantation, highlighting where current data are insufficient and specifying the types of studies needed to advance the field. It summarizes gaps across pharmacologic therapy (antiresorptive and osteoanabolic agents), fracture risk assessment tools (BMD, HR-pQCT, FRAX, and AI-based models), surgical and biochemical management of mineral metabolism (cinacalcet vs. parathyroidectomy, bone biopsy), and the skeletal impact of immunosuppressive regimens. For each clinical area, the table contrasts the present limitations of the evidence base with proposed study designs, such as adequately powered randomized trials and prospective cohorts, to better define strategies that reduce fracture risk and improve skeletal outcomes in KTRs

Area / Clinical question	Current evidence gap	Type of study needed
Fracture end points for antiresorptive therapy (bisphosphonates, denosumab) in KTR	RCTs powered for BMD only; fracture risk reduction unproven in transplant populations	Adequately powered RCTs with fracture as primary end point
Optimal timing: prophylactic vs. monitoring-triggered pharmacologic therapy	No head-to-head trial in de novo KTR	RCT stratified by fracture risk
Predictors of heterogeneous early BMD trajectories	Risk factors distinguishing bone losers from nonlosers poorly defined	Prospective cohort with serial BMD and metabolic profiling
HR-pQCT fracture risk thresholds specific to KTR	Mostly general-population reference ranges; no validated transplant-specific cut-offs	Large prospective study linking HR-pQCT to incident fractures in KTR
FRAXplus vs. FRAX ± BMD for fracture prediction in KTR	Data in KTR are limited; no robust evidence that adding BMD to FRAX improves prediction performance in this population.	Prospective validation cohort with long follow-up
Osteoanabolic therapies (teriparatide, romosozumab) in KTR	One small RCT for teriparatide; romosozumab small retrospective series (*n* < 20) show safety but lack control groups.	Controlled trials with BMD and fracture end points
AI-based fracture risk models in KTR	Exploratory only; no prospective validation in transplant populations	Multicenter prospective cohort studies
Cinacalcet vs. parathyroidectomy: skeletal outcomes and fracture risk	Biochemical efficacy established; skeletal/fracture benefit not demonstrated for either	RCT powered for fracture or BMD outcomes Head-to-head cinacalcet vs. parathyroidectomy
Role of bone biopsy/histomorphometry in post-transplant care	Clinical decision thresholds undefined; no data on when biopsy changes management	Prospective studies defining utility of histomorphometry
Long-term skeletal impact of mTOR inhibitors	Conflicting signals; no long-term controlled fracture data	Long-term follow-up from immunosuppression trials

BMD, bone mineral density; FEA, finite element analysis; FRAX, fracture risk assessment tool; HR-pQCT, high-resolution peripheral quantitative CT; KTR, kidney transplant recipient; mTOR, mechanistic target of rapamycin; RCT, randomized controlled trial.

Robust randomized controlled trials remain needed to determine the effects of antiresorptive and anabolic therapies on hard clinical outcomes such as fractures, graft survival, and patient mortality, as available evidence continues to rely largely on surrogate end points.

### Conclusion

BMD loss during the first post-transplant year is rapid, clinically relevant, and driven by immunosuppressive exposure, persistent CKD-MBD and altered bone remodeling. Modern diagnostic tools, particularly HR-pQCT and FEA, reveal cortical deterioration and microarchitectural compromise that often precede measurable changes in aBMD. Integrating imaging modalities with biochemical markers may improve phenotyping of post-transplant bone disease and support individualized management.

Moving forward, standardization of microarchitectural assessments and validation of imaging-based prediction models of failure load and fracture risk will allow rigorous evaluation of the effects of pharmacologic interventions, preferably through adequately powered randomized controlled trials. These measures could further improve long term skeletal outcomes in KTRs.

## Disclosure

MP declared honoraria for lecturing from AstraZeneca, Boehringer Ingelheim, CSL Vifor; consulting fees from GlaxoSmithKline. PE declared consulting fees and research grant from CSL Vifor; HZ received research grants from Pharmocosmos and Novo Nordisk, consulting fees from Astra-Zeneca, Kedrion, Menarini, Pierre Fabre, Pharmacosmos, honoraria for lecturing from Falk Foundation, Menarini, Pharmacosmos. AK received research grants from CSL Vifor and Otsuka, consulting fees from Amgen, Argenx, AstraZeneca, Boehringer Ingelheim, CSL Vifor, Delta4, GlaxoSmithKline, Novartis, Novo Nordisk, Otsuka, Roche, Sobi, and Walden Biosciences. CU declared no competing interests.

## Data Availability Statement

All data discussed in this article are available in the public domain.

## Author Contributions

The conceptualization and methodology was done by all authors; Writing of the original draft by CU and AK; Writing, review, and editing by all authors. All authors read and approved the final manuscript.
